# Do moments of inattention during study cause the error-speed effect for targets in recognition-memory tasks?

**DOI:** 10.3758/s13423-024-02475-7

**Published:** 2024-02-26

**Authors:** Anne Voormann, Constantin G. Meyer-Grant, Annelie Rothe-Wulf, Karl Christoph Klauer

**Affiliations:** Department of Psychology, University of Freiburg, Freiburg, Germany

**Keywords:** Recognition memory, Error-speed effect, Continuous models, High-threshold models, Attention

## Abstract

The error-speed effect – characterized by a decreased performance in a second recognition task for stimuli that elicited fast error responses in a first recognition task – has so far been predominantly interpreted as evidence for the existence of misleading memory information. However, this neglects a possible alternative explanation, namely that the effect may instead be caused by moments of inattention during study. Here, we introduce a manipulation that allowed us to distinguish between words from the study phase that participants most certainly paid attention to and those they did not. We hypothesized that if moments of inattention cause the error-speed effect, this effect should disappear when considering only targets that verifiably received attention during study. However, our results (*N* = 89) suggest that this is not the case: The error-speed effect still occurs for targets that participants attended to during study and thus indeed seems to be caused by misleading memory evidence rather than by moments of inattention during study.

An ongoing debate in the memory literature concerns the origin of errors in standard recognition paradigms. The main question revolves around whether some of these errors are caused by misleading mnemonic information or, alternatively, whether all of them are the result of incorrect guessing when decisive mnemonic information is absent (see, e.g., Starns, 2021; Starns et al., 2018; Starns & Ma, 2018). This issue is closely linked to the question of whether the discrete-state *two-high threshold model* (2HTM; Snodgrass & Corwin, 1988; for an illustration, see Figure 1a) can adequately account for human decision-making in various recognition-memory tasks (see, e.g., Bröder & Schütz, 2009; Kellen & Klauer, 2014, 2015; Malejka et al., 2022; Meyer-Grant & Klauer, 2021). Recently, an empirical finding known as the *error-speed effect* (Starns et al., 2018; Voormann et al., 2021, 2023) has received some attention due to its putative implications regarding this controversy. Indeed, the effect seems to suggest that memory sometimes misleads, which challenges a key assumption of the 2HTM, namely that recognition errors result exclusively from incorrect guessing.^1^

**Fig. 1:**
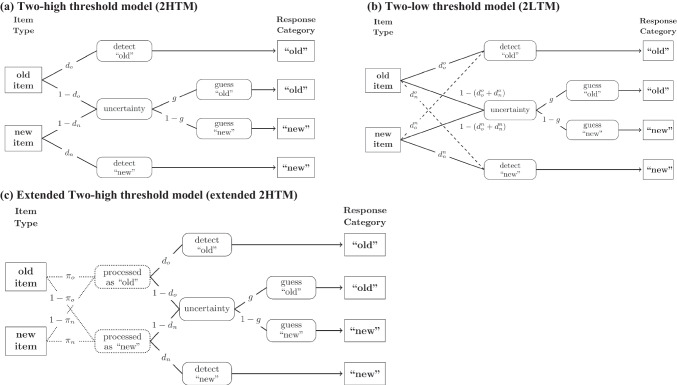
*Illustration of the two-high threshold model (a)* *and the* *two-low* *threshold* *model* *(b)* *and* *the* *extended* *two-high* *threshold* *model (c). Note.* The models show the discrete processing states (in curved boxes) and their conditional probabilities along each path (e.g., $${d}_{n}, {d}_{o}^{o})$$ for old and new items in a single-item task that lead to the response categories “old” and “new.” Dashed lines indicate the additional probabilities of incorrect detection states for old ($${d}_{n}^{o})$$ and new items ($${d}_{o}^{n}$$) in the 2LTM. Dotted lines indicate the additional processing states and their probabilities (e.g., $${\pi }_{o}$$, $${\pi }_{n}$$) of the extended 2HTM

In its original form, the error-speed effect refers to an interesting phenomenon that can be observed when participants first complete a *single-item old/new* (SION) task followed by a *two-alternative forced-choice* (2AFC) task. In each trial of a SION task, participants specify for one item at a time whether this item was previously studied (i.e., an old item) or not (i.e., a new item). In each trial of a 2AFC task, on the other hand, participants are presented with an old–new item pair (i.e., two items, one old and one new) and are instructed to select the item which they believe is more likely to be the old one. The error-speed effect alludes to the observation that the likelihood of participants providing a correct response in a 2AFC task depends on how quickly they responded incorrectly to one of those items in the preceding SION task (Starns et al., 2018). More specifically, the faster an item is falsely classified in the SION task, the less likely it is that a pair including this item will be correctly solved in the 2AFC task.

Continuous dynamic recognition-memory models (such as the *diffusion model*; Ratcliff, 1978) offer a compelling explanation for this effect. These models assume that recognition decisions and the speed with which they are made depend directly on the strength of underlying *memory signals* (see, e.g., Van Zandt, 2000). That is, the stronger the memory signal elicited by an item, the more *familiar* it appears to the decision maker (e.g., Morrell et al., 2002). Moreover, such an increase in the subjective evidence for the present item being old not only increases the probability of an “old” response, but also leads to these responses being given more quickly (Starns et al., 2018).

An important feature of continuous recognition-memory models is that the memory signal of any given item is assumed to be stochastic in nature and hence needs not always be aligned with the item’s true status (i.e., whether it is old or new; see, e.g., Ratcliff, 2014; Starns & Ma, 2018). For instance, an old item may sometimes – albeit on average less often than a new item – elicit a relatively weak memory signal, making it appear rather unfamiliar to the decision maker and thus resulting in an incorrect response. In such a case, an old item elicits misleading memory evidence.^2^ It then follows from the above that items eliciting a subjectively conclusive but objectively misleading memory signal (e.g., old items that appear very unfamiliar) will also tend to lead to faster incorrect responses in a SION task compared to items that elicit more ambiguous memory signals (Starns et al., 2018).

For 2AFC tasks, continuous models of recognition memory usually postulate that responses are determined by comparing the memory signals of both presented items with each other (see, e.g., Kellen et al., 2021). Thus, the more misleading the memory signal of any given item, the more likely an incorrect response occurs. The weaker the memory signal of the old item, for example, the higher the likelihood that this item appears even less familiar to the decision maker than the actual new item, leading to the new item being mistakenly selected.

By combining the above-outlined mechanisms for SION as well as 2AFC recognition tasks, one finds that continuous models can predict the error-speed effect as long as one is willing to uphold the assumption that multiple memory signals associated with the same item are at least correlated between different points in time. In other words, if an item appears relatively (un)familiar during an initial SION task, it is likely to continue appearing relatively (un)familiar during a later 2AFC task.

In contrast to continuous models, discrete-state models reject the notion that decision makers can directly access the memory signals elicited by encountered items and instead assume that decisions are mediated by discrete mental states. That is, items may be detected as “old” or as “new”, or they may enter a state of uncertainty (Kellen & Klauer, 2018; see also Figure 1), which leaves decision makers with no choice but to guess a response option in a SION task. These states are each entered with a certain probability (such as ${d}_{n}$, the probability to detect a new item as new and ${d}_{o}$, the probability to detect an old item as old in Figure 1a). However, once a mental state is reached, response probabilities depend only on that state and not on the underlying strength of the memory signal (Chen et al., 2015; Province & Rouder, 2012; Rouder et al., 2014).

Although there is no direct link between the underlying memory-strength signals and the response probabilities, some discrete state models, such as the *two-low threshold model* (2LTM; Starns, 2021; Starns et al., 2018; see Figure 1b), are consistent with the error-speed effect. This can be attributed to the fact that these models allow the decision maker to enter an *incorrect* detection state (see dashed lines in Figure 1b), which corresponds to the erroneous belief that an old item is new or vice versa. Furthermore, response speed is determined by the sum of process times associated with the different mental states that contribute to a response (see, e.g., Klauer & Kellen, 2018). Assuming that responses out of a state of (incorrect) detection are faster than (incorrect) guesses (Heck & Erdfelder, 2016; Province & Rouder, 2012), the model predicts that items that elicit fast error responses in SION tasks are more likely to have been incorrectly detected than items that elicit slow error responses. Consequently, these incorrect detected items should then also lead to a higher likelihood of an incorrect response in a subsequent 2AFC task.^3^ However, the 2LTM was only recently developed and is not the most common model in the recognition memory literature.

On the other hand, the above-mentioned 2HTM – arguably one of the most widely used discrete-state models of recognition memory (e.g., Bröder & Schütz, 2009; Snodgrass & Corwin, 1988) – has recently been criticized on the ground of being unable to predict the error-speed effect. This is because, unlike the 2LTM, it does *not* allow for the possibility of *incorrect* detection (see Figure 1a). Therefore, errors arise solely from incorrect guessing decisions out of a state of uncertainty (Snodgrass & Corwin, 1988). However, since this entails that all errors result from the same underlying mental state, the speed of an erroneous SION response cannot predict response accuracy in the 2AFC task, as it is not indicative of systematic differences in subjective mnemonic information. Thus, the traditional version of the 2HTM is generally unable to account for the error-speed effect.

However, as discussed by Voormann et al. (2021; see also Meyer-Grant & Klauer, 2021), it is possible to extend the 2HTM by allowing for the possibility that nominally old items sometimes function as new items, and new items sometimes function as old items for reasons elaborated on below (see Figure 1c). In consequence, items can end up in incorrect detection states just like in the 2LTM. Thus, as long as responses out of a detection state are again assumed to be faster than guesses, the extended 2HTM is likewise able to account for the error-speed effect by virtue of its indirect allowance for incorrect detection states (if, e.g., an old item enters the branch for new items, it can be incorrectly detected as new).

The 2LTM and the extended 2HTM are actually mathematically equivalent when considering their predictions for response frequencies (Meyer-Grant & Klauer, 2021). However, the two models differ in their psychological interpretation, which is crucial for the present study. In the 2LTM, items can be incorrectly detected due to genuinely misleading mnemonic information. In contrast, the extended 2HTM still dispenses with the notion of misleading memory evidence and hence only indirectly permits nominally old items to enter the branch of the processing tree that is usually associated with new items and nominally new items to enter the branch usually associated with old items (compare Figure 1b and Figure 1c).

But this raises the question whether there is a psychologically plausible explanation for such a cross-contamination between the branches for new and old items (i.e., nominally old items sometimes functioning as new ones and nominally new items sometimes functioning as old ones) while retaining the assumption that there is no misleading mnemonic information. Although coincidental similarity to an old item could be considered a possible reason why some new items are mistaken for being old, there is a particularly compelling explanation for why old items sometimes function as if they were new: Participants may simply process some items inadequately during the study phase.^4^ Suppose, for example, that a participant has failed to pay (sufficient) attention to a certain number of items. Obviously, these items were never encoded into memory and therefore can only be treated as new items in a later test phase. Simply put, an item that was never committed to memory can also not be recognized.

Interestingly, this alternative explanation has not yet been empirically investigated – at least not to our knowledge. In the present work, we thus aim to fill this gap by trying to replicate the error-speed effect for old items that participants have verifiably attended to during study. For this purpose, we implemented an attention check procedure, which required participants to recall the studied words after their presentation in the study phase.

If the error-speed effect disappears for old items with a successful attention check, it would suggest that previous occurrences of the effect are likely caused by old items that were never properly encoded into memory and hence functioned exactly like new items. If, however, we still replicate the error speed effect for old items that participants have clearly attended to during study, this would further corroborate the notion that misleading memory signals must underlie the effect.

## Methods

All materials to run the experiment as well as the data and analysis scripts are available on the Open Science Framework (https://osf.io/kv5j8/).

### Participants

We aimed for a sample size of 85 valid data sets and ended up collecting data from 96 participants. To evaluate the required sample size, we considered the smallest effect size in Voormann et al. (2021). Using the effect size *d*_*z*_ = 0.36, an alpha of .05, and a power of .95 in a one-sided paired t-test, *N* = 85 is required as the sample size.

From the total sample, two participants had to be excluded due to technical problems and an additional five participants did not fulfill the inclusion criteria defined as a difference between the *hit* (“old” response to an old item) and *false alarm* (“old” response to a new item) rate to be higher than .1. This resulted in 89 valid datasets. The final sample (66 females and 23 males) had a mean age of 21.35 years (SD = 3.03), age ranging from 18 to 36 years.

Data collection took place online. We checked every 3 weeks for the number of complete datasets. Data collection finished at the earliest point in time at which the number of complete datasets was equal to or exceeded the intended sample size. We recruited participants via the online platform Sona-Systems University of Freiburg. Participants had to be aged between 18 and 45 years, speak German fluently, and needed normal or corrected-to-normal vision. Additionally, we asked participants to complete the study in a quiet place using a laptop or personal computer. For complete participation, participants received partial course credit.

### Material

Stimuli were randomly drawn from a wordpool of 639 neutral German nouns provided by Lahl et al. (2009). Words were four to eight letters long with medium valence and low arousal. All words were approximately equally frequent in spoken German, as indicated by the log frequency ratings obtained for each word via WordGen (Duyck et al., 2004).

### Procedure

After providing informed consent to participate in the study, participants encountered two types of cycles during the experiment: one practice cycle and two experimental cycles. The purpose of the practice cycle was to acquaint participants with the different tasks and the structure of the experiment.

Each cycle combined a study phase and a subsequent test phase. During the study phases, participants were instructed to memorize blocks of four words presented sequentially at screen center for 2,000 ms (with an inter-stimulus interval of 100 ms). After each block, a free recall task required participants to list the four words they had just seen by typing them on the keyboard. This recall task served as the attention check that allowed us to identify those studied words that participants definitely attended to during study. For practice cycles, the study phase consisted of seven blocks with four words each (28 words in total). For experimental cycles, participants worked on 27 blocks of four words (108 words in total). In both practice and experimental cycles, a blank screen of 500 ms separated two blocks. The first and the last block of each study phase served to buffer primacy and recency effects and were discarded for the test phase.

Each test phase consisted of alternating SION and 2AFC blocks. For the SION task, participants categorized words from the study phase (henceforth also referred to as *targets*) and new items (henceforth also referred to as *lures*) as either having been studied before (being “old”) or not (being “new”) by pressing the respective response key (“M” for old words and “Y” for new words on a German QWERTZ keyboard). Each word was presented along with the “ALT” and “NEU” (German for “OLD” and “NEW”) key assignment below the stimulus until the participant’s response. After an inter-trial interval of 100 ms the next stimulus appeared.

For the 2AFC task, each trial consisted of a target and a lure presented next to each other on the screen. Participants had to identify the actual target by pressing either the “Y” key if they believed they had studied the left word before or the “M” key if they thought they had studied the right word. In each trial, both words appeared separately on-screen for 1,000 ms (starting with the left word) before being presented together until the participant’s response. Labels below word pairs indicated the response mapping. The position of the target (left/right) was counterbalanced within each block. After an inter-trial interval of 100 ms, the next stimulus pair appeared.

Each SION block consisted of 20 trials including ten targets and ten lures. The first SION block of each cycle contained four additional warm-up trials that consisted of two targets drawn from the first four words studied (i.e., the primacy and recency buffers) and two lures. Warm-up words were discarded for the 2AFC task and will therefore also be excluded from data analyses. Additionally, the sequence of targets and lures was randomized within each block.

Each 2AFC block contained ten trials consisting of words presented in the preceding SION block. In general, 2AFC trials can combine items that fall into one of four categories depending on whether the item is old or new, and on whether the previous single-item recognition response to this item was correct or not. The categories in question are hits, false alarms, *misses* (“new” responses to old items), and *correct rejections* (“new” responses to new items). For data analysis, however, the critical trials were those that paired an item to which the participant had previously responded correctly with an item to which the participant had previously responded incorrectly. Thus, those combinations (hit & false alarm; miss & correct rejection) were preferred. Other combinations (hit & correct rejection; miss & false alarm) were used only when none of the items required to form one of the preferred pairs was available anymore.

In total, participants completed two series of the SION task and the subsequent 2AFC task within the practice cycle. In each experimental cycle, ten series of the SION task and the subsequent 2AFC task were administered leading in sum to 408 single-item trials and 200 2AFC trials. To ensure that participants were prepared to respond, each SION block started with a keypress of the “M” and “Y” key as well as a countdown lasting 3,000 ms. Prior to each 2AFC block, a press of the space key was required.

### Analyses

To test the hypothesis, we pre-registered to compare the 2AFC performance between fast and slow target errors for attended targets using a paired *t*-test (see https://osf.io/z6gkh).^5^ Therefore, we categorized errors based on the individual median error speed and separately for targets and lures (see also Starns et al., 2018; Voormann et al., 2021). SION error responses that were faster than the respective median reaction time (RT) were categorized as fast errors, whereas error responses slower or equal to the respective median RT were considered as slow errors.

In addition to the pre-registered tests, we computed a hierarchical logistic regression with the correctness of the 2AFC trials (correct: 1; incorrect: 0) as the dependent variable. To keep this analysis conceptually close to the analyses via *t*-tests, we included the predictor 2AFC trial type coding the kind of word pair (hit & false alarm [H–FA]: 1 vs. miss & correct rejection [M–CR]: -1). Additionally, we considered separately for target and lure errors the predictors error speed (z-standardized and log-transformed RT) and correct speed (z-standardized and log-transformed RT) in the SION task as well as their pairwise interactions. In order to obtain separate estimates for the coefficients of error speed, correct speed, and their interaction for both 2AFC trial types, we first generated two additional dummy-coded variables: target error (H–FA: 0 vs. M–CR: 1) and lure error (H–FA: 1 vs. M–CR: 0). Next to the fixed-effects factor 2AFC pair types, we then also included the fixed-effects two-way interactions between target error and error speed, target error and correct speed, lure error and error speed, lure error and correct speed, as well as the fixed-effects three-way interactions between target error, error speed, and correct speed and between lure error, error speed, and correct speed. This statistical model is equivalent to a model that includes the three fixed effects 2AFC trial types, error speed, and correct speed, as well as all their possible interactions, but has the advantage of allowing us to interpret the tests associated with the coefficients in a more straightforward manner. Analogous to Starns et al. (2018), we normalized RT distributions by taking the logarithm and standardized the log RTs separately for correct target, incorrect target, correct lure, and incorrect lure responses using the respective mean but the overall standard deviation of target and lure RT distributions due to small cell numbers.

Furthermore, we included crossed random effects for participants, lure words, and target words (Judd et al., 2012). We fitted the hierarchical logistic regression with a maximal number of 100,000 iterations for conversion, the “bobyqua” optimizer, and a maximum tolerance of 0.01. Applying the “keep it maximal” principle proposed by Barr et al. (2013), we conducted a backwards selection for determining the random-effects structure. For feasibility reasons, we first conducted three separate backwards model selection procedures including only one of the three random-effect factors (i.e., participants, lure word, or target word). Each of those three selection procedures started with the respective maximal random-effect structure, that is, all random-effect factors and their correlations were initially included. If a model failed to converge or showed a singular fit, we systematically reduced the random-effects structure based on the least incremental explanatory value (in terms of variance accounted for). However, exclusion did not violate the principle of marginality and the correlations between random effects were always excluded first. The maximal random-effects structures for each of the three random-effects factors that converged and led to a nonsingular fit were then combined and the resulting model served as a starting point for a final model selection procedure containing all three random-effect factors. This was accomplished by yet another backwards selection adhering to the same principles as the previous ones. The *p*-values for fixed effects in the final model were determined via likelihood ratio tests.

## Results

### Data preparation

Following Starns et al. (2018), we excluded trials with responses faster than 400 ms or slower than 8,000 ms in the SION task (1.4% or trials). We also excluded trials comprising items that were not correctly recalled during study (5.0% of trials). Furthermore, only the critical trials of the 2AFC task were included in the analyses; that is, trials that combined a word that was incorrectly detected in the SION task with another one that was correctly detected (i.e., H–FA pairs and M–CR pairs). Of responses to old words, 29.8% were misses, and 15.6% of responses to new words were false alarms. This resulted in an average number of 55.4 (*SD* = 23.5) M–CR pairs and 28.9 (*SD* = 18.0) H–FA pairs per participant.

### Inspection of the error-speed effect

The pre-registered paired *t*-test revealed a significant error-speed effect even for targets to which participants had verifiably paid attention during the study phase (see Table 1). As predicted, fast target errors in the SION task led to less accurate responses in the subsequent 2AFC task (*M* = 67.4%, *SD* = 46.9) compared to slow target errors (*M* = 71.4%, *SD* = 45.2).

**Table 1:** Mean correct responses (in %) and standard deviations for the two-alternative forced-choice (2AFC) task as well as mean and standard deviations of response times (in ms) in the single-item old/new (SION) task separately for fast and slow responses on error and correct trials in the SION task with the results of the paired-samples *t*-tests

Performance in SION task		Fast	Slow	*t*(88)	*p*	*d*_z_ [95% CI]
Target error (miss)	acc	67.4 (46.9)	71.4 (45.2)	2.01	.047	0.24 [0.001; 0.47]
	rt	772 (196)	1456 (791)			
Lure error (false alarm)	acc	77.0 (42.1)	77.0 (42.1)	0.10	.922	0.01 [-0.20; 0.22]
	rt	848 (235)	1633 (914)			
Target correct (hit)	acc	80.1 (40.0)	74.0 (43.9)	3.62	< .001	0.39 [0.17; 0.60]
	rt	753 (137)	1333 (752)			
Lure correct (correct rejection)	acc	74.0 (43.9)	65.0 (47.7)	7.30	< .001	0.78 [0.54; 1.03]
	rt	732 (139)	1314 (811)			

Although not relevant for our research question but for completeness, we also tested for the occurrence of the error-speed effect within lures and the correct-speed effect within targets and lures. A correct-speed effect is consistent with all recognition memory models: Fast correct responses in the SION task should lead to a higher accuracy in the subsequent 2AFC task than slow correct responses. While the error-speed effect for lures did not reach statistical significance, the correct-speed effect occurred for both targets and lures (see Table 1). In other words, fast-correct responses in the SION task resulted more frequently in correct 2AFC performance than slow-correct responses.

### Hierarchical logistic regression

Table 2 shows the results for the final hierarchical logistic regression model.^6^ As can be seen, the fixed-effects of 2AFC trial type, as well as the two-way interactions between lure error and correct speed, target error and correct speed, and target error and error speed were statistically significant. For the 2AFC trials of type H–FA (i.e., a lure error trial) there was an increased probability of a correct response compared to trials of type M–CR (i.e., a target error trial). Importantly, faster error responses to targets (but not to lures) led to a decreased probability of a correct response, attesting the error-speed effect for targets. Additionally, faster correct responses generally led to an increased probability of a correct response, demonstrating the correct-speed effect. The interaction between correct and error speed generally did not contribute significantly to the prediction.

**Table 2:** Regression weights (estimates and standard error), the likelihood ratio test statistic, and corresponding *p* values for the fixed effects of the hierarchic logistic regression

	Estimate (SE)	${\chi }^{2}$(1)	*p*
2AFCType	0.25 (0.06)	34.22***	< .001
TargetError $\times$ LogErrorRT	0.13 (0.04)	11.88***	< .001
TargetError $\times$ LogCorrectRT	-0.20 (0.04)	22.83***	< .001
TargetError $\times$ LogErrorRT $\times$ LogCorrectRT	-0.05 (0.03)	2.56	.109
LureError $\times$ LogErrorRT	-0.07 (0.05)	1.89	.169
LureError $\times$ LogCorrectRT	-0.24 (0.05)	25.27***	< .001
LureError $\times$ LogErrorRT $\times$ LogCorrectRT	-0.01 (0.05)	0.01	.908

*** *p* < .001

## Discussion

In the present experiment, we found evidence for the error-speed effect for targets as indicated by a reduction in 2AFC accuracy for items that led to fast compared to slow error responses in a previous SION task even when considering only old items that were correctly recalled during the study phase and that therefore definitely received attention during study. This effect was supported by a significant pre-registered paired *t*-test. Additionally, this result was also corroborated by a hierarchical logistic regression that treated error response time as a continuous predictor and accounted for potential variability between participants and items (i.e., the presented words).

Taken together, these results speak to the idea that misleading memory signals cause the error-speed effect, as they run counter the predictions of the extended 2HTM, which has been proposed specifically to reconcile the 2HTM with previous instances of the error-speed effect (Voormann et al., 2021). This model conceptualizes only those old items as old to which participants actually paid attention during study, whereas otherwise old items are assumed to function as if they were new. In the present study, however, we still observed the error-speed effect for targets, although we restricted our analyses to old words that were correctly recalled in the study phase. These items must have been processed and encoded into memory during study and, thus, they should always act as old items in the rationale of the extended 2HTM. Due to this restriction, the extended 2HTM is identical to the traditional 2HTM for this subset of items, as all errors must result from the same underlying uncertainty state. Accordingly, the speed of incorrect responses in the SION task should not have been predictive of the accuracy in the 2AFC task.

Thus, extending the 2HTM by incorporating an attention-based process that determines which old item actually functions as old is not sufficient for enabling the model to account for the present data. However, although our manipulation ensured that items were perceived and actively processed in working memory during study, there is still the possibility that not all items included in the analysis were in fact encoded into long-term memory. Thus, an alternative interpretation of the additional path of the extended 2HTM might be that only studied items that are successfully encoded into long-term memory serve as old items during the recognition test, whereas old items not transferred to long-term memory might still function as lures in the SION task.

But notwithstanding such objections, our results highlight – at the very least – that the 2HTM has an undisclosed limiting condition regarding what is required for there to be truly *high* thresholds. In the present work we have demonstrated that high thresholds conflict with experimental results not only for nominally old items (i.e., items that were presented during study) but also for *subjectively* old ones (i.e., items that were perceived and actively processed by the decision maker). That said, future work should clearly consider the question of whether there is a certain point of memory consolidation beyond which memory can no longer be misleading.

Although questioning the high-threshold assumption, our results do not necessarily refute the basic notion of discrete mental states mediating underlying memory-strength signals. The 2LTM (Starns, 2021; Starns et al., 2018; see Fig. 1b), for instance, is generally consistent with the error-speed effect as encountered in the present study. At first glance, this might appear surprising given the fact that the 2LTM is mathematically equivalent to the extended 2HTM – at least if only response frequencies are considered (Meyer-Grant & Klauer, 2021). But other than the extended 2HTM, the 2LTM is not predicated on the high-threshold assumption, which consequently allows for an alternative psychological interpretation. More precisely, while both models incorporate the possibility to enter an incorrect detection state (see Figs. 1b and 1c), the 2LTM differs from the extended 2HTM in that such a state can be induced by any studied item and is not restricted to targets not attended to during study, which reflects the possibility for genuinely misleading mnemonic information. Thus, the 2LTM is able to predict an error-speed effect even for old items that received attention during study.

Nevertheless, the probability of entering an incorrect detection state is likely to be lower for old items to which participants paid attention during study than for those to which they did not. For this reason, the 2LTM would actually predict that the error-speed effect decreases in analyses that consider only old items attended to during study compared to an analysis that includes all studied items. This prediction is also in line with continuous dynamic recognition-memory models: Targets that received no attention during study should evoke a relatively weak memory signal because there is no systematic subjective memory evidence suggesting that these items are old. Therefore, one should not only expect an increase in the relative frequency of “new” responses for these items, but also that these incorrect responses are given more quickly (Starns et al., 2018).

And indeed, by comparing 2AFC performance between fast and slow errors when including all studied items, we descriptively found a more pronounced error-speed effect. The effect size of the error-speed effect when including all old items amounted to *d*_*z*_ = 0.29 with a 95% CI of standardized effects of [0.06; 0.53]. This was higher than the reported *d*_*z*_ = 0.24 with a 95% CI of standardized effect of [0.001;0.47] of the pre-registered analysis that included only old items attended to during study. However, since these assessments were made post hoc and more extensive data are required for an appropriate inferential treatment, this finding must be interpreted with caution. Therefore, future research should further investigate the precise impact of targets not attended to during study on the size of the error-speed effect.

Coming back to the implications of the error-speed effect for recognition decisions, our present findings strengthen the general conclusion that recognition errors do not seem to be solely the result of incorrect guesses. Although inattention to items during study might slightly increase the effect size of the error-speed effect, the effect is still present when controlling for attention – a finding that is consistent with the 2LTM as well as dynamic recognition theories, both of which account for the effect via misleading memory evidence. Those results also inform the structure that is necessary for other memory models such as process models, for example, MINERVA 2 (Hintzman, 1988) or SAM (Gillund & Shiffrin, 1984), to be able to account for the observed effect: Allowing responses based on misleading memory evidence, be it discrete or continuous, is an essential part in recognition decisions. Overall, our results therefore clearly support the notion that the error-speed effect reflects misleading memory evidence and is not caused by moments of inattention during study.

## Footnotes

1 It should be noted that, in general, it has long been known that memory can be highly misleading (see, e.g., Deese, 1959; Loftus & Hoffman, 1989; Roediger & McDermott, 1995). However, these well-established effects rely on experimental paradigms that are specifically designed to induce false memories. This contrasts with the central question investigated in the present study, which is whether systematically misleading memory information can manifest itself without such manipulations.

2 At this point, it may be helpful to clarify that mnemonic information is considered to be misleading whenever the memory signal is comparatively weak for an old item leading to an incorrect new response or comparatively strong for a new item leading to an incorrect old response.

3 Similar to continuous models, this prediction relies on the assumption that the discrete mental states evoked by the same item at different points in time are *not* independent.

4 As an interesting side note, this assumption would also explain the presence of a curious pattern previously observed in studies examining the error-speed effect, namely that the effect appears to be larger for errors associated with old items than for errors associated with new items (Voormann et al., 2021) – a result that we in fact also replicate in the present study. Note, however, that according to Starns et al. (2018), continuous dynamic recognition memory models likewise predict such an asymmetry.

5 As it is good statistical practice, we used arc-sine transformed 2AFC performance, which was a deviation from our pre-registered analysis plan. This transformation changed some values numerically but not the associated statistical conclusions.

6 This model included random intercepts for participant and lure word. Furthermore, the model included by-participant and by-lure random slopes for 2AFC trial type and the two-way interactions between target error and correct speed, as well as a by-participant random slope for the two-way interaction between target error and error speed, but no correlations between random effects.
